# A case study of partnership in practice: challenges and insights in the development of an academic-community coalition “The Migrant Health Community Research Network”

**DOI:** 10.1186/s40900-025-00828-7

**Published:** 2025-12-20

**Authors:** Jessica Carter, Felicity Knights, Yusuf Ciftci, Kathryn Mackey, Eltayeb Hassan, Ada Jusic, Isra Al-Sharabi, Anna Deal, Nuria Sanchez Clemente, Alison Crawshaw, Sally E. Hayward, Darlington Faijue, Nathaniel Aspray, Natalie Elkhier, Philippa Harris, Rosita Chia-Yin Lin, Beatriz Morais, Sarah Tizzard, Oumnia Bouaddi, Farah Seedat, Sally Hargreaves

**Affiliations:** 1 Migrant Health Research Group, Institute for Infection and Immunity, School of Health and Medical Sciences, City St Georges, University of London, London, UK; 2 Wolfson Institute of Population Health, Queen Mary’s University of London, London, UK; 3 Migrant Health Community Research Network, London, UK; 4 Refugee Council, London, UK; 5 London School of Hygiene and Tropical Medicine, London, UK; 6 Southwark Refugee Communities Forum, London, UK; 7 City and Hackney Public Health Team, London, UK; 8 University College London Hospital, London, UK; 9 St. George’s University Hospitals NHS Foundation Trust, London, UK; 10 Department of Public Health and Clinical Research, Mohammed VI Center for Research and Innovation, Rabat, Morocco; 11 Mohammed VI International School of Public Health, Mohammed VI University of Sciences and Health, Casablanca, Morocco

**Keywords:** Migrant health, Patient engagement, Participatory research, Health inequities, Research priorities, Co-production

## Abstract

**Background:**

Participatory research (PR) approaches are increasingly prioritised globally, particularly in the UK, where many funders have emphasised meaningful collaboration with those with lived experience to improve health research relevance, translation and impact. Despite this, marginalised groups, such as migrants, remain underrepresented in research, perpetuating health inequities. Migrants, who comprise 16% of the UK population, face systemic barriers to engagement, including distrust, hierarchical academic structures, and lack of inclusivity. PR approaches offer a collaborative framework for empowering migrant voices, balancing research and action, and fostering trust to address these disparities. This case study describes the development of the Migrant Health Community Research Network (MHCRN), a collaboration between migrant community groups, individuals, academics and health professionals from the Migrant Health Research Group, City St Georges University of London (MHRG). **Aim** MHRG sought to develop a sustainable model for engaging migrants in health research through a co-created network addressing power imbalances and ensuring inclusivity.

**Methods:**

MHRG adopted PR principles, reflecting on the groups collective experience of prior engagement practices and systematically reviewing best practices. A five-phase approach was used: (1) defining agenda and aspirations through group reflection; (2) identifying concerns and challenges through internal reflection and community consultation; (3) conducting community exploratory workshops with co-facilitation; (4) establishing initial network structure based on collectively agreed principles; (5) co-developing a lived experience advisory panel.

**Findings:**

The process has resulted in the following outputs: the “Migrant Health Community Research Network” (MHCRN) with guiding principles (equity, diversity, respect, decolonisation, empowerment, community), a network steering group (LEAP, Lived Experience Advisory Panel), community-based collaborations embedding migrant voices at all research stages, capacity building through training and peer researcher roles, and award-winning projects. Key challenges identified include structural inequalities, funding limitations, and institutional barriers. Opportunities emerged around trust-building, shared leadership, and sustainable relationship development.

**Conclusions:**

While the MHCRN represents an important step in embedding meaningful collaboration, challenges persist. This initiative highlights the need for systemic change in academia to support equitable partnerships and inform similar innovative initiatives. By prioritizing non-tokenistic engagement and co-production, the MHCRN sets a foundation for sustained, impactful collaboration to address health inequities and inform policy and practice.

**Plain English summary:**

Many health research projects fail to involve people they aim to help, especially marginalised groups such as migrants. This often results in research that is neither relevant nor useful. We recognised that the way we have worked with people with lived experience of migration could be improved. Our aim was to create a way to work together long-term so that migrant voices are heard and acted on from the development of research ideas all the way to the delivery and sharing of findings. We decided after talking to many researchers, migrants, community organisations and health care professionals that a migrant health research network that brought together all these voices through relationship building and creative ways of working and sharing ideas would be a good way to do this. We established the Migrant Health Community Research Network (MHCRN) which brings together people living in the United Kingdom with lived experience of migration from all over the world, healthcare professionals working in the National Health Service (NHS) and researchers from City St George’s University of London to improve migrant health. Our network was built step-by-step, starting with open conversations with migrants, healthcare professionals and researchers about challenges they had experienced in involving migrant communities in research and how these could be overcome. We then ran a workshop to shape our vision and agreed on shared principles. The network aims to be long-term, inclusive, and led by those with lived migration experience. Although there are ongoing challenges, this process has taught us valuable lessons about working together in a meaningful way.

## Take home messages

**Sustained relationship building and transparency are essential**: Meaningful engagement of migrants in health research requires moving beyond tokenistic involvement to true sustained partnership and shared leadership to create safe spaces which avoid “data extraction” and instead facilitate authentic participation.

**Creative methods, community leadership and practical support (childcare, accessible venues) can enhance engagement:** Cultural sensitivity, people with lived experience in leading roles, and the use of arts-based and other innovative approaches can bridge gaps, enrich dialogue, and create inclusive spaces for collaboration and diverse participation.

**Structural challenges require systemic change:** Traditional academic structures and funding mechanisms can impede sustainable community engagement, requiring institutional change through the recognition and proactive response to the resource implications (both time and funds) of doing participatory research well.

**Reflexivity is crucial**: Academic researchers must continuously examine their own roles and privileges, ensuring that where possible power imbalances are addressed and avoided.

## Introduction

### Background and context

Public and patient engagement and the use of participatory research approaches have rightly moved up the agenda globally. In the United Kingdom the National Institute of Health Research has produced the INVOLVE criteria to provide clear guidance for researchers on meaningful ways of working with those with lived experience to improve quality, relevance and impact of health research [1]. It is increasingly recognised that public engagement with research and participatory approaches are essential if research is to meet the needs of those it seeks to help. For example, collaborative working using innovative participatory research methodologies such as co-production can improve the gap in translation of research to real-world settings as the views of end users are incorporated from the outset [2–5].

While there is a growing appreciation for the importance of patient and public involvement and engagement in academia, with some funders making it a mandatory component for funded projects, implementation remains inconsistent. Despite this paradigm shift, public engagement can often be exclusive, tokenistic and not representative of the target population [6]. Marginalised groups, including those with lived experience of migration, remain under-represented in health research, and face substantial barriers to participation. The barriers to engagement stem from multiple sources, including the inherent power dynamics within academic institutions, systemic undervaluation of experiential knowledge compared to academic credentials, and deeply rooted institutional practices (embedded within the wider hostile environment) that often fail to build trust with communities [7–9]. This is especially the case with the most marginalised such as undocumented migrants, newly arrived individuals, and migrants who don’t speak any English. Another concern relates to small and “extractive” type studies with limited impact, resulting in further lack of trust and engagement from the community in the research process [10]. For example, the persistent under-representation of diversity in clinical trials generates a gap in the data that potentially skews medical innovation towards interventions which lack efficacy and safety information for minority populations. [11, 12] As this type of study does not capture the lived experiences of migrants, there are multiple consequences of continuing with this approach. These can include: migrant voices not being heard, migrants’ research priorities being either misunderstood or misrepresented, the knowledge produced being limited and potentially biased, migrant health needs remaining unmet with resultant health inequalities, and persistence of social exclusion which in itself is an injustice [13–16]. This exclusion creates a self-perpetuating cycle where research priorities miss community needs, and interventions fail to address the most pressing concerns of these populations, with implications for health equity.

Public engagement and participatory research methodologies and the way they are applied exist along a continuum ranging from informing and consultation to collaboration and community-owned research. This is depicted in Arnstein’s classic ladder of participation and the International Association for Public Participation’s spectrum of participation [17, 18]. The theoretical foundations of participatory approaches draw from multiple traditions, including Freire’s critical pedagogy as well as feminist and decolonising methodologies [19–21]. At their core, participatory research approaches challenge traditional power hierarchies in knowledge creation through the positioning of community members as co-researchers and valuing lived experience as a form of knowledge [22, 23]. This approach recognises that those who experience health inequities possess essential expertise about the causes, consequences and potential solutions [22, 23]. We recognise that participation and participatory are often over-used terms and have expanded on our interpretation and use in this manuscript in box 1 below.

**Table:** 

Box 1: Our definition of participatory research
We recognize that “participatory research” is often used broadly to describe various forms of community engagement. For this manuscript, we distinguish between participatory and consultative approaches. While some of our research group’s methods include some traditional qualitative techniques (workshops, interviews and focus groups), what makes the network’s aim participatory rather than consultative is a shift in power dynamics.
We define our participatory approach as research where migrant community members are not just consulted or involved as data sources, but are proactively involved in the research process through shared decision-making in priority setting; research design; collaborative analysis and interpretation of findings; joint problem-solving when challenges arise; and collective ownership of research outputs and dissemination. This contrasts with research where participants inform predetermined research questions but do not shape the fundamental direction or interpretation of the work.

### Migrant health context

Within migrant health research participatory approaches must navigate complexities. Migration itself is recognised as a social determinant of health that can lead to health inequities [24]. International policies are clear that communities should be involved in health research, yet this is not always the case [25]. The challenge lies in operationalising participatory principles within communities that may be characterised by extreme diversity, varying legal statuses, language barriers, and previous experiences of research exploitation

The current national context makes addressing these challenges through inclusive research approaches increasingly urgent. Over 16% of the UK population was born abroad, and due to the current geo-political climate, economic changes and the ever-increasing threat of climate change migration is increasing, despite the hostile environment present across Europe [26–28]. Migrant health likewise has gained traction as an independent research field. The COVID-19 pandemic highlighted the stark reality of the health inequities migrant groups face in the UK; with migrants experiencing higher infection rates, more severe illness, and greater economic impacts while simultaneously being underrepresented in research and vaccination programmes [28–31]. These health challenges are multi-faceted. Migrants are recognised to experience high rates of depression, anxiety and post-traumatic stress disorder, often exacerbated by experiences of discrimination social isolation and uncertainty about legal status [32–35]. Access to culturally competent mental health services remains limited, with language barriers and lack of cultural understanding among healthcare providers creating additional obstacles to care [35]. Physical health inequities also exist. Migrants face significant barriers to accessing primary healthcare, exclusion from NHS services based on immigration status, complex charging policies, and fear of immigration enforcement in healthcare settings [36]. Preventative healthcare uptake is consistently lower among migrant populations, contributing to later presentations of infectious and some non-communicable diseases with resultant poorer health outcomes dependent on country of origin and duration of residence [30, 31]. The hostile environment policies implemented across the UK have created additional structural barriers to health access, fundamentally undermining trust between migrant communities and health services [36]. These systemic barriers operate alongside individual-level challenges including language barriers, unfamiliarity with healthcare systems, and previous traumatic experiences in countries of origin [36].

Recent years have seen growing adoption of creative research methodologies and frameworks in the migrant and inclusion health field in response to a recognition of a lack of diversity in study participation and the recognition of the importance of the lived experience voice. These offer valuable models for engaging marginalised populations in health and include amongst others; a UK charity, Groundswell, creating solutions to end homelessness who have developed a model of peer-led research “Experts by Experience” and advocacy with training and ongoing support structures who work alongside research groups building relationships with communities on the basis of shared experience [37, 38]. A recent publication led by an international group for the World Health Organisation who developed a comprehensive framework for refugee and migrant health research in the WHO European Region, emphasising community engagement at its core to support participatory, interdisciplinary, and inter-sectoral projects [14]. This framework explicitly addresses the need for coordinated effort to advance migration research through participatory approaches, providing methodological guidance that has influenced policy development across multiple countries [39]. The ABCD national Research Partnership in Australia exemplifies a large scale long-term partnership, where Australian Indigenous health research has pioneered community advisory models that establish formal mechanisms for Aboriginal and Torres Strait Islander people to have meaningful involvement in research governance; and in this case using participatory action research approaches to improve the quality of primary health care available to Indigenous people in 140 health centres across five states and territories [40]. In the UK the ATD Fourth World organisation has developed participatory research approaches specifically focused on including people living in extreme poverty, and their model demonstrates methods for engaging populations who face multiple barriers to participation, emphasising long-term relationship building, peer support and capacity building through the development of “people’s universities” where community members develop research and advocacy skills [41]. Alternative and more inclusive research approaches that incorporate arts, storytelling, and music as communication methods are also becoming more common in health research, in particular with marginalised groups [42–45]. These innovative approaches aim to maximise engagement while respecting and incorporating cultural practices which are particularly important for migrant communities.

A “nothing about us without us” philosophy, successfully employed by other inclusion health groups is particularly relevant for migrant health research [46]. This approach demands engagement with those having lived experience of migration from the earliest stages of research conceptualisation through to implementation and translation. We can learn a lot from the experiences of other groups such as those outlined above, and while these provide valuable insights migrant health presents specific challenges that require adapted approaches. Unlike relatively stable communities of place or single-issue focus areas, migrant communities are characterised by extraordinary diversity in terms of countries of origin, migration trajectories, legal statuses, languages, cultural practices, and socioeconomic backgrounds. This diversity creates both opportunities and challenges for participatory research. As we outline below, building on both the experiences of other groups and our own learning, our network differs from some existing approaches in several key ways, including: significant time spent on relationship-building and transparency about past research limitations before pursuing specific research objectives; recognising extreme diversity as an asset through using the shared migration experience as common ground; explicit discussion of institutional barriers from the outset; and aiming for co-facilitation from inception. This paper aims to contribute to the field of participatory research and migrant health through sharing our practical experience and methodology, as well as the co-produced knowledge gained through developing a sustainable participatory migrant research partnership with highly diverse, potentially transient, and systematically marginalised communities who face particular barriers to traditional approaches to research engagement.

### Study aims

The aim of this paper is to outline the co-development of the Migrant Health Community Research Network, a community-academic collaboration of people with lived experience of migration in the UK and the City St George’s Migrant Health Research Group (MHRG) with the shared aim of producing meaningful collaborative migrant health research. It is essential that groups working in this space are continuously reflective and share learning, as it is only through sharing experiences of both the successes and critically the challenges of participatory migrant health research that we will progress.

## Methods

### Setting and context

The City St George’s Migrant Health Research Group is a group of clinical and non-clinical academics who work closely with public health and policy colleagues, the charity sector and community organisations, and essentially, people with lived experience of migration to bring about improvement in migrant health both in the UK and internationally. The group takes a holistic, participatory, solutions-focused approach to health inequities facing migrant groups and utilises a diverse range of research approaches with the aim of influencing policy, improving clinical practice and ultimately reducing health inequities.

The group has always aimed to conduct research centred on lived experience, including embedding patient and public involvement and engagement (PPIE) at its core, publishing a review of participatory research approaches to migrant health interventions and establishing an advisory board of experts by experience to have oversight of the group’s projects in 2018 [47]. However, the group increasingly recognised that there were limitations with the established approach to engagement. These included project-focused involvement, involvement late in the research cycle, problems of potential top-down dynamics and perpetuating power imbalances, and the lack of a sustainable cross-project engagement model. Further, migrants are a highly heterogeneous group, with diverse backgrounds, experiences, needs and capabilities. As such, it was felt that the diversity of cultures, languages and needs of distinct migrant groups required specific consideration to develop a genuinely meaningful and inclusive process. We felt a sustainable network would facilitate diverse voices to propose and shape research priorities and enable the building of relationships based on mutual trust and respect to directly address potential power imbalances.

### Positionality and reflexivity

The MHRG recognised that reflexivity would be essential for the development of a migrant health academic-community partnership, as partners navigate complex power relations and the recognition of different forms of knowledge [48, 49]. Those in academic or clinical positions (MHRG) must continuously examine how their institutional authority, professional and educational status, and often privileged social locations shape their assumptions, approaches, and interactions [50]. This reflexive practice involves acknowledging that while academic expertise is valuable, it exists within hierarchical structures that have historically marginalised migrant communities’ knowledge and experiences with roots in a colonial past [51]. Through ongoing critical self-reflection, the MHRG can better understand how their positionality influences the collaborative space and work to create conditions where community expertise is centred and valued. For migrant community members, their positionality often includes navigating multiple intersecting experiences of marginalisation while holding deep expertise about their communities’ needs and strengths. In contrast to traditional hierarchies, academic clinical professional statuses may carry less weight when navigating community networks, while community members’ experiential knowledge is essential for successful collaboration [48, 49]. Remaining cognisant of these complex positionalities is key to allow space for discussion about how the coalition can structure itself to promote genuine power-sharing and ensure community knowledge shapes its direction and priorities [48, 49].

### Funding and ethical considerations

The group secured £10,000 funding from Research England (St George’s University of London Participatory Research England Call) to explore and develop the idea of a sustainable migrant health academic-community partnership model using participatory approaches. Ethics approval was not required for this network development activity. Consent was obtained from MHCRN members for writing up this case study, which was produced collaboratively on behalf of the group.

### Network development approach

**Fig. 1:**
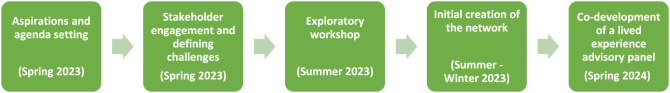
Five-phase participatory approach timeline

In collaboration with migrant organisations and community members with lived experience of migration the aim was to work towards a sustainable model of facilitating meaningful and collaborative migrant health research. A five-phase participatory approach was envisaged: Fig. 1.

**Phase 1: Defining Agenda and Aspirations** As a research group over a series of meetings (May 2023- June 2023) we defined a need for a formalised, longitudinal approach to working with people with a lived experience of migration. We intentionally sought out and discussed examples of best practice from our previous systematic review on the topic [47] and shared learning about the experiences of other institutions which have successfully established longstanding collaborative networks with inclusion groups [14, 37–41], but worked to avoid defining the structure or function of our network so that this could be collaboratively developed.

**Phase 2: Identifying Concerns and Challenges** The MHRG engaged in a process of reflection on our previous work and the concerns and challenges we had developed or read about in the literature surrounding participatory research with inclusion groups. Through a series of reflective sessions using post-sticks, graffiti walls, peer-led group discussion and culminating in a SWOT analysis (strengths, weaknesses, opportunities, threats) [52] in May-June 2023 the research group (consisting of 12 clinical and non-clinical academics at early, mid and established career research stages) documented their concerns and challenges regarding effective participatory research and engagement with migrant groups. This internal reflection was followed by several months of one-to-one meetings (May-July 2023) with local community groups and individuals previously involved in research projects. Recruitment was initially through those groups previously involved in research projects with the MHRG, followed by word-of-mouth as well as JC reaching out to community groups found online via email. The only criteria were that individuals or groups had lived experience of migration to the UK and an interest in migrant health. For accessibility reasons meetings were held either in-person (in the setting of the community member’s choosing—mostly community halls) or online, if this was preferred, and led primarily by JC with support from SH and FK. Meetings were primarily held in English, but where required community members brought interpreters from their own groups. These were unstructured interview style conversations, exploratory in nature to allow for open discussion and transparency between communities and the academic group to understand their previous experiences, concerns and challenges.

**Phase 3: Exploratory Workshop** We invited all those who had been involved in the conversation to date to an exploratory workshop to further refine our agenda and collaboratively explore approaches that would enable us to overcome these concerns and challenges. Taking on board feedback from the community members, we worked together with Southwark Refugee Communities Forum as partners to run an exploratory workshop on 7^th^ of July 2023. This partnership between the academic group (MHRG) and an organisation run by and for those with lived experience allowed shared ownership of how the workshop could best fulfil the objective of enabling a safe space to co-develop a network focused on participatory migrant health research. This importantly included hosting the workshop in a community space in a diverse area of London and not a university building with inherent hierarchical connotations.

Workshop activities included:

- An introduction to co-production principles and the participation ladder with interactive activities by a researcher with lived experience
- Identification of research priorities through a group prioritisation and voting exercise
- Co-development of the network structure, communication strategies and guiding principles using world café methodology [53]
- Design of network identity through a collaborative art exercise led by artist with lived experience

It is important to note here that this workshop was held in-person and led in English due to the nature of the community hall and the activities planned. To address issues with accessibility, future events were planned to be hybrid where possible and with planned online sessions for those members unable to leave home or travel. Participants were able to bring young children to the event to ensure inclusivity to those with childcaring responsibilities, and care was taken to match those people less confident in English with peers who could interpret. Facilitation was done slowly to allow effective and accurate interpretation. It was agreed again that where possible future events would be held in multiple languages to maximise participation and inclusivity.

**Phase 4: Initial Network Creation** Following the workshop, we worked collaboratively to establish the initial network structure based on our collectively agreed principles. We built on the learning we had done so far, prioritising engagement and community led agenda setting, ensuring flexibility of meeting formats and locations, providing practical support to attend (childcare, reimbursement, travel and food) and focusing on sustainable relationships rather than transactional interactions.

**Phase 5: Co-development of Lived Experience Advisory Panel** The establishment of a steering group named the “Lived Experience Advisory Panel” or LEAP was a milestone for the network. An expression of interest form was created and advertised to the network who collectively agreed that the steering group or panel should consist of members with lived experience of migration, an active interest in migrant health and research and represent a diverse range of views and lived experiences.

We were guided by the principles of reflexive thematic analysis to conduct collaborative analysis of reflective session notes, community consultation summaries, and workshop outputs [54]. Academic researchers undertook initial coding to identify patterns related to engagement challenges, opportunities, and potential solutions, which were then reviewed and refined through discussion with the community members who have also co-authored this paper to ensure findings resonated with lived experience and authentically represented diverse perspectives [54].

The roles of the community members and the roles of the academic group members were complementary throughout each phase. The MHRG academic members primarily supported the administrative functioning of each phase with shared decision making about timings, geography and other practical aspects (e.g. food etc) with community members. Community members roles included both leading and co-facilitation of activities at workshops, leading discussions and democratic voting around key decisions such as the network structure, name, logo, research priorities, communication methods and timings and ideal format and composition of an advisory panel.

## Results

### Phase 1 results: agenda and aspirations definition

The research group successfully established consensus around the need for a sustainable, longitudinal approach to migrant engagement that moved beyond project-specific involvement in the form of a collaboration or network. Key aspirations identified included:

- Creating genuine power-sharing mechanisms
- Establishing trust-based relationships
- Developing culturally responsive research approaches
- Ensuring sustainable funding models
- Building community capacity and leadership

### Phase 2 results: challenges and concerns identification

The group reflection process revealed substantial challenges across multiple domains. These ranged from practical considerations such as language barriers and digital access to deeper structural issues around power dynamics and representation. One particularly powerful reflection came from a team member who noted:

*We must acknowledge that we’re working against a system that privileges quick, hierarchical structures within universities and the NHS, without real funding or recognition for community engagement.* (MHRG team member)

The internal group reflection as outline above was complimented by one-on-one meetings with community members. These conversations revealed additional challenges as well as echoing those outlined by the group, including fears about exploitation and concerns about the sustainability of engagement efforts. As two community members reflected:

*Too often we are asked to share our stories, but we never see what happens afterward. We need to know that our involvement will lead to real change. *(community member with lived experience of migration)

*We know the groups that don’t work respectfully with migrants, we talk to each other you know, word gets around *(community member with lived experience of migration)

These initial reflections are summarised in Table 1 below

**Table 1:** Identified challenges to meaningful migrant health participatory research

**Methodological challenges**	
Representation and inclusivity	*Failure to capture a broad range of views beyond the “usual participants”* *Challenges in representing diverse migrant populations (recognising “migrant” is a broad term)* *Difficulty engaging those not hard to reach but “easy to ignore” inclusion health groups* *Boards/committees not truly representative of target populations* *Lack of diversity in participants (same kinds of people consistently participating)*
Communication and Participation Barriers	*Language barriers limiting meaningful contribution* *Digital barriers excluding certain populations* *Format, timing, and location considerations not tailored to participants’ needs* *Silence or awkwardness in participatory settings* *Fear of lack of skills and knowledge, apart from lived experience* *Lack of adjustments for inclusivity* *Infrequency of board meetings meant losing connection and lack of sense of belonging*
**Ethical Concerns**	
Power Dynamics	*Top-down power dynamics within research processes* *Power imbalances between researchers and participants* *Power hierarchies within migrant communities themselves* *Researcher-driven agendas rather than community priorities*
Exploitation and Tokenism	*Research benefiting researchers without genuine community involvement* *Tokenistic inclusion without meaningful engagement* *Extractive research practices not benefiting communities* *Participants misunderstanding potential benefits (e.g., expecting healthcare or visa assistance)*
Cultural Sensitivity	*Making assumptions about community definitions, preferences, or needs* *Discussing sensitive topics (disease burden in migrant populations) insensitively* *Bringing up traumatic past events without appropriate support* *Not recognising or avoiding colonial approaches to research* *Causing offense through inappropriate framing or discussion*
**Implementation Challenges**	
Sustainability and Follow-through	*Inability to act on identified priorities or needs* *Lack of sustainable engagement strategies* *Constraints from traditional research thinking and approaches* *Difficulty establishing consensus on how to move forward*
Misalignment of Expectations	*Differences in expectations between researchers and participants* *Research not being helpful for either researchers or participants* *Participants expecting direct benefits (healthcare, legal assistance) that researchers cannot provide*

The process also generated preliminary solutions for overcoming these challenges and a vision began to develop for the network and what it would aim to achieve (outlined in Table 2 below). As one community member acknowledged:

*It’s important to acknowledge where things have gone wrong in the past, but equally important to show commitment to working collaboratively towards better engagement methods. (*community member with lived experience of migration)

**Table 2:** Potential approaches to meaningful migrant health participatory research

**Community-Centred Research Design**	Develop research questions and methodologies collaboratively with community members Ensure research addresses community-identified priorities Use participatory research methods that share power with participants
**Inclusive Engagement Strategies**	*Provide language support through provision of interpreters* *Offer multiple participation formats (digital, in-person, written)* *Schedule activities at times and locations convenient for participants* *Compensate participants appropriately for their time and expertise*
**Ethical Safeguards**	*Establish clear boundaries and expectations at the beginning of engagement* *Provide appropriate support for discussions of sensitive topics* *Ensure research findings benefit the communities being studied* *Build in mechanisms for community feedback throughout the research process*

### Phase 3 results: exploratory workshop outcomes

The exploratory workshop brought together researchers, facilitators with lived migration experience and 35 migrant community members from 29 different community groups. The event was co-hosted by City St Georges, MHRG and Southwark Refugee Forum led by EH, and co-facilitated by members with lived experience of migration (EH, YC, AJ). There was live illustration of the event by an artist herself with lived experience of migration (AJ). The leading of this workshop and subsequent co-facilitation of activities by those with lived experience was key to fostering trust and ensuring open dialogue.

The research prioritisation exercise revealed the breadth and depth of health issues concerning migrant communities with over thirty health topics put forward as a priority by participants. The three top priorities were agreed as culturally competent mental healthcare, culturally competent preventative healthcare and equitable access to health care, and it was agreed that future events should focus on these priorities.

A key moment was the creation of the network’s guiding principles through the world café approach (listed below box 2) which emphasised inclusion, respect, and equity.

**Table:** 

Box 2: Co-produced network guiding principles
Equity and health for all
Diversity
Community at heart
Respect
Decolonisation and challenging discrimination
Working together
Empowerment

Participants expressed the importance of feeling genuinely heard and valued through the participatory facilitation methods used and during the day as a whole. One participant, emphasised the impact of shared leadership:

*It’s really important to have someone leading who has been in my shoes. It makes people feel reassured and open. *(community member with lived experience of migration)

*‘I don’t like to go where there is no lived experience. It’s really, really important to have someone leading/facilitating to have that experience. It means you can be open. When you tell them ‘I have been there’ it makes them feel a bit more reassured that their season may pass. They have been in my shoes and know what I am going through’.* (community member with lived experience of migration)

Another participant reflected on the diversity of the event:

*I have never been to a workshop where so many communities were represented, the common experience was being a migrant it didn’t matter where you were from *(community member with lived experience of migration)

**Fig. 2:**
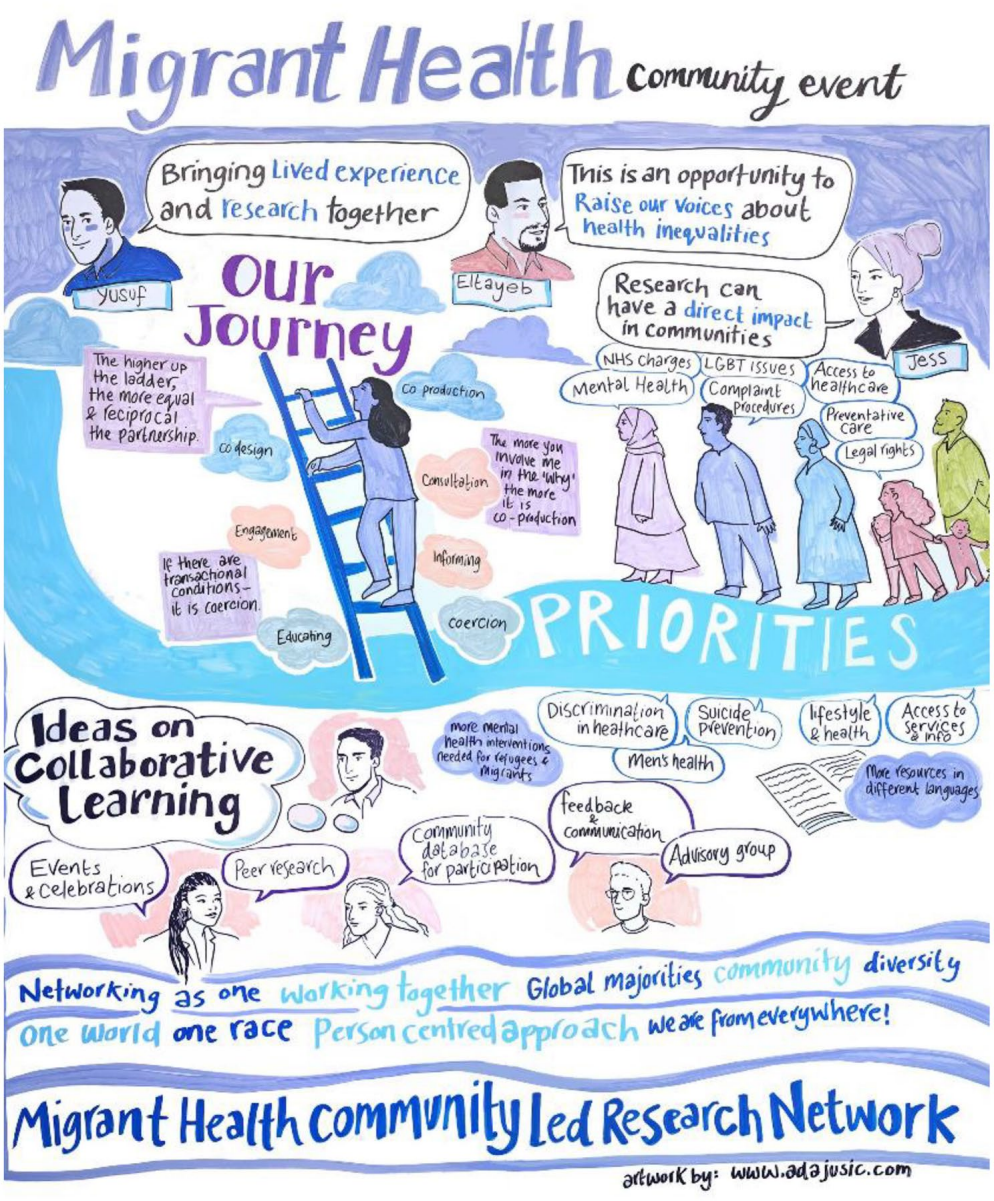
A visual representation of the migrant health exploratory workshop by Ada Jusic (herself a person with lived experience of migration)

This sentiment reflected the workshop’s success in promoting ownership and alignment with participants’ lived experiences. A visual representation of the workshop can be seen in Fig. 2.

Four core themes emerged from the exploratory workshop:

1. **Trust Building:** The importance of acknowledging past limitations and investing time in relationship-building before pursuing research objectives proved crucial. This approach, while time-consuming, created a foundation for authentic collaboration.
2. **Structural Changes:** Traditional academic structures often impede meaningful engagement. Universities need to develop new frameworks for community partnership, including appropriate payment mechanisms and recognition of lived experience expertise.
3. **Sustainability:** Long-term sustainability requires addressing both funding mechanisms and institutional barriers. Current project-based funding models don’t adequately support ongoing community engagement.
4. **Cultural Competence:** The diversity within migrant communities requires careful attention to cultural dynamics and power relationships, including those within communities themselves.

It was agreed that next steps should build on these themes and the guiding principles and include the formal creation of the network, a follow-up event focusing on preventative migrant health care and an introduction to policy and the formation of an advisory board to embed lived experience voices in the day-to-day work of the research group.

### Phase 4 results: initial network creation

**Fig. 3:**
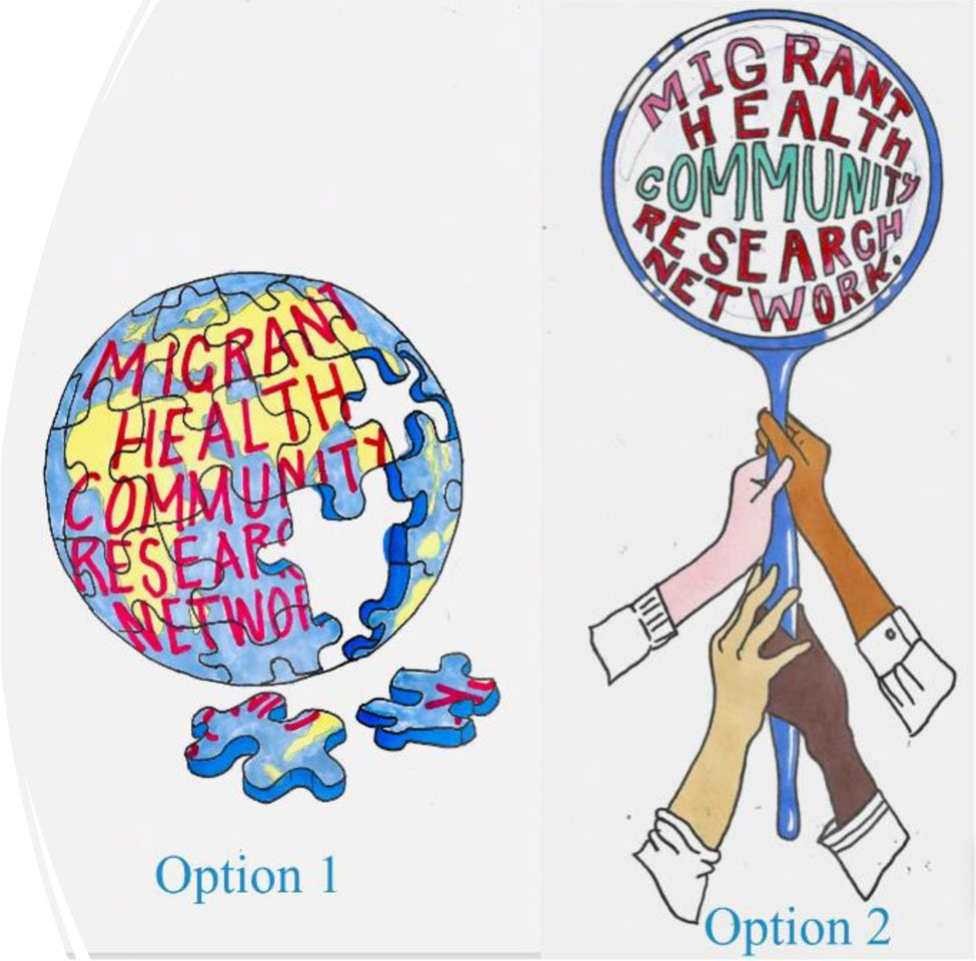
Initial logo designs for the Migrant Health Community Research Network

The network’s identity began to take shape. Participants collaboratively decided on a draft name the “Migrant Health Community Research Network”, (MHCRN), finalised the guiding principles outlined above and voted on the initial logo designs created during the workshops (Fig. 3 below). Early activities focused on health and policy issues raised during the workshop, including workshops on accessing healthcare services, diverse models of delivering migrant healthcare, culturally competent preventative healthcare and rights. Events were planned with an emphasis on flexibility to accommodate participants’ availability and preferred formats, ensuring a welcoming and accessible environment for all. Efforts to sustain momentum included regular communication through newsletters via a network email address, informal catchups every four-six months on Zoom, which were instrumental in maintaining engagement, promoting a sense of network ownership and building relationships, and in person workshops every nine to twelve months with a focus on a particular health issue important to the network as well as a capacity building exercises and networking opportunities.

**Fig. 4:**
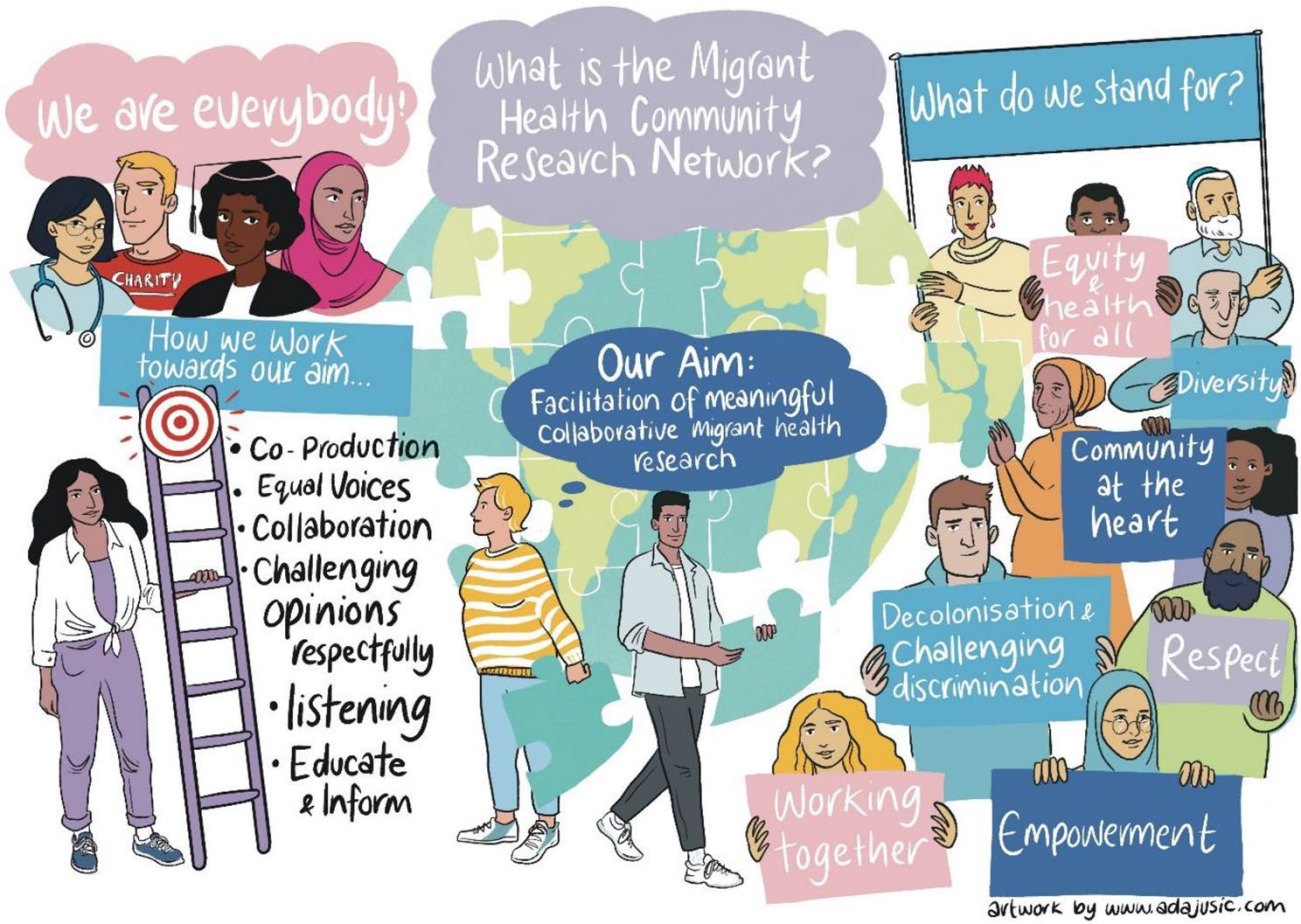
Visual representation of the Migrant Health Community Research Network

**Fig. 5:**
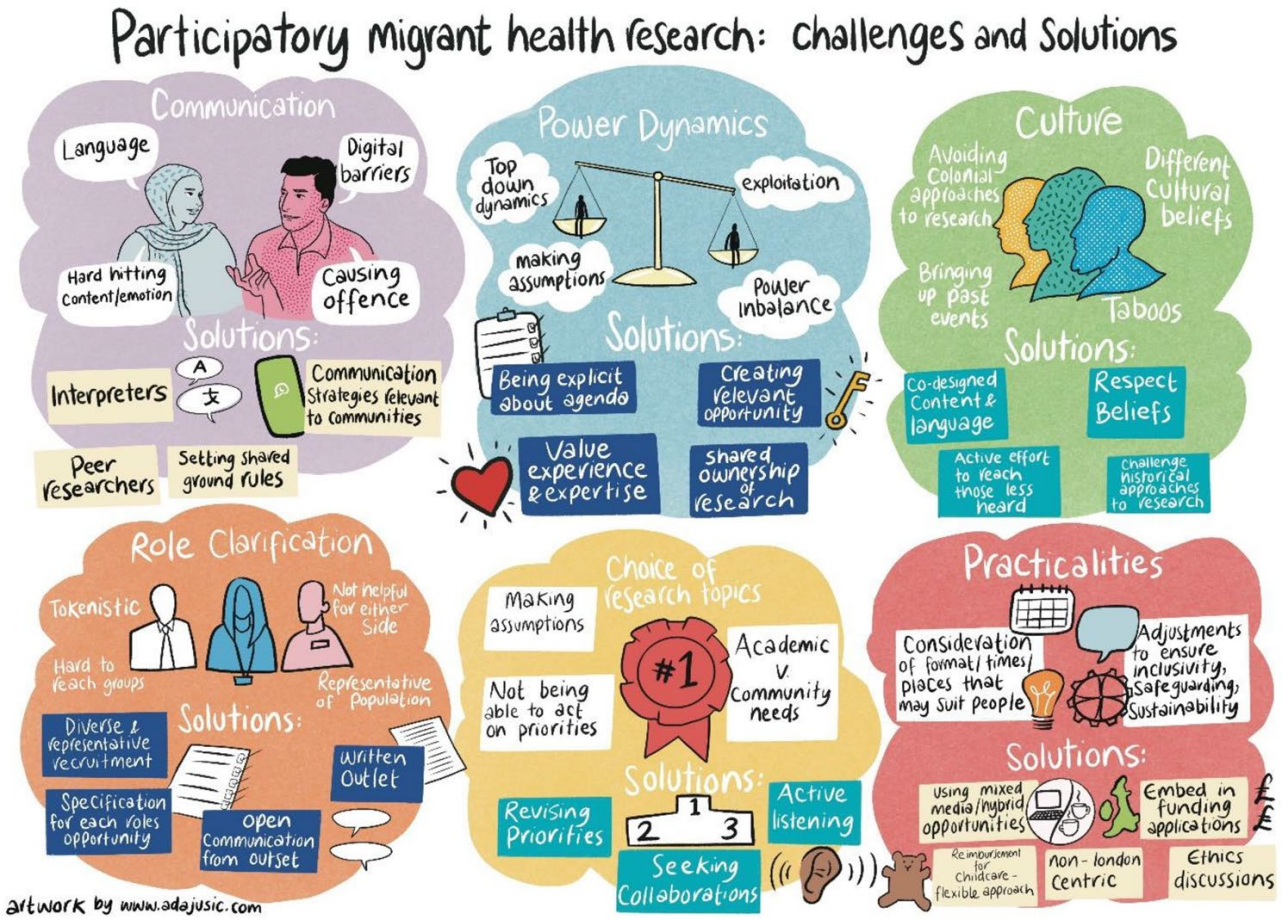
Participatory migrant health research: challenges and solutions

Infographics were co-produced by the network to allow for visual representation of what we stand for as well as the challenges and collectively determined potential solutions to meaningful migrant health participatory research. (Figs. 4 and 5 below).

Although challenges remained key successful elements of this network should be highlighted. These include: the proactive prioritisation of relationships over transactions, network members have reflected that they feel like partners rather than subjects. The importance of support mechanisms for participation including provision of childcare, reimbursement, travel and food. Meeting on the community’s terms in community settings, creating a website (https://www.migranthealthnetwork.org/) and using WhatsApp to communicate and to share opportunities has shown respect for community members time and ways of working and has increased engagement and created a sense of identity. Maintaining transparency about challenges has helped build trust and shared understanding of limitations to this way of working but also promoted a collective approach to solutions.

At this stage, the majority of MHCRN activities were coordinated by academics within the MHRG, with collaboration from a facilitator with lived experience of migration who was contracted with funds from the MHRG to support with planning and co-facilitate events. While all events and initiatives are planned in collaboration with the broader MHCRN, the heavy administrative role played by the academics (in addition to their other responsibilities and dependent on funding within research grants) was noted as both a threat to the sustainability of the network and an opportunity to further enhance the ownership and decision-making power of the network itself. With this in mind, recent grant funding has included allocated resource for an MHCRN member to share this administrative role for the next 3 years including running and updating the website, coordinating communications and event organisation as well as maintaining connection with the community and increasing membership.

#### Co-development of a lived experience advisory panel and future vision

Our Lived Experience Advisory Panel (LEAP) was formed in Spring 2024 and comprises 12 migrant representatives, who were selected based on their interest in engaging further, while actively ensuring the diversity of the group in terms of language spoken, country of origin, gender, age, sexual orientation and disability. LEAP meets quarterly with the MHRG and feeds into research projects conducted with the group, inputs into the strategy and function of the MHCRN and ensures that its activities remained grounded in community needs. Regular meetings provide a forum for reflective practice, identifying areas for improvement, and addressing challenges as they arise. A shared aspirations and commitments document created by LEAP reflects our approach to this way of working (see Appendix 1). Although newly established, LEAP aims to act as an advisory board to the MHRG, by providing regular inputs on research projects and outputs, through review of funding proposals, grants and papers as well as regular meetings in which researchers present on specific topics and seek inputs and guidance from LEAP. LEAP also supports the logistics and functioning of the network, including providing advice to the facilitators on how the network should function, and topics for future meetings and events. In the future, it is envisaged that LEAP will take a larger role here, enabling the MHCRN to attract its own funding. Through involvement with LEAP, it is envisaged that individuals will gain practical skills and experience in leadership, partnership building and research.

## Network achievements

The MHCRN has reached its two-year anniversary, has over 70 members representing over 40 communities, and has celebrated achievements, awards and collaborations with significant impact summarised in Table 3 below. As well as fostering a participatory research culture across the MHRG, these include the above co-produced infographics allowing visual representation of values, challenges, and collectively determined solutions to meaningful migrant health participatory research. The production of a report for the British Red Cross in collaboration with peer researchers from the network, creation of a policy document with the UKHSA on refugee and asylum seeker’s access to healthcare, being awarded research image of the year by City St George’s for the visual representation of the network created by Ada Jusic above and one of the members winning lived experience research project of the year at the Lancet Public Health Conference 2024.

**Table 3:** MHCRN achievements

Research project	MHCRN input	Output
**Creation of Migrant Health Community Research Network**	Community consultation Network workshops	Research England participatory research funding 2023 City St George’s University Image of the Year 2024: *A visual representation of the migrant health community research network* School for Academic Primary Care conference: Oral presentation 2024 Academy of Medical Sciences funding awarded 2024
**Co-producing a model of asylum seeker and refugee collaboration in regional health policy and practice: a qualitative applied research methodology**	Community consultation Participatory workshops	Guidance for the United Kingdom Health Security Agency to form a health community advisory board for asylum seeker and refugee health policy. Lancet abstract 10.1016/S0140-6736(24)01983–4)
**A framework to guide the commissioning of healthcare services for people seeking asylum in England: a qualitative co-production study** (British Red Cross commissioned work)	Co-production workshops Peer researchers Member presentation: Dr Isra Al-Sharabi	Policy report UKHSA abstract regional meeting Lancet abstract (10.1016/S0140-6736(24)01989–5) Best Oral Presentation for Lived Experience Involvement at The Lancet Public Health Science Conference 2024
**Migrant Health Catch-UP!** **A clinical decision support tool for migrant health checks in UK primary care.**	Formation of an academic-practice-community coalition Two participatory workshops Peer researchers leading interviews	Research England participatory research funding 2024 WHO Compendium: *Promoting the health of refugees and migrants: experiences from around the world* 10.1016/S0140-6736(24)01989–5 Wellcome Trust funding for school for primary care research PhD 2024 -28. School for Academic Primary Care: Oral presentation 2025
**Labour migration exploitation**	Lived Experience Advisory Panel consultation during grant inception and development Peer researchers	National Institute for Health Research funding 2025–2027 Wellcome trust discovery grant 2026–2031

## Ongoing challenges

While the network has achieved significant progress, the process has however revealed significant and persistent structural barriers within academic institutions, including:

- Complex bureaucratic requirements conflicting with community needs.
- Funding structures poorly aligned with community engagement needs (e.g. use of specific vouchers/reimbursement for transportation etc).
- Lack of established mechanisms for employing peer researchers.

The ongoing challenges we face which threaten the sustainability of the MHCRN

1. **Sustainable Funding:** The lack of long-term funding mechanisms continues to challenge the network’s sustainability, particularly regarding compensation for community partners.
2. **Institutional Constraints**: Traditional academic timeframes and requirements often conflict with community-centred approaches, requiring constant negotiation and adaptation.
3. **Representation**: Ensuring broad representation across diverse migrant communities remains an ongoing challenge, particularly regarding engaging the most marginalised groups.

## Sustainability and power-sharing

While the sustainability of the network remains an ongoing challenge, we are taking deliberate, system-level steps to ensure the Migrant Health Community Research Network (MHCRN) becomes self-sustaining. These efforts focus not only on securing funding, but also on redistributing power, strengthening governance, and embedding participatory practices across the research ecosystem. Key actions include:

1. Transitioning Governance to the Lived Experience Advisory Panel (LEAP): We aspire to LEAP progressively sharing responsibility for the network’s governance, administration, and strategic fundraising. This transition represents a shift from academic stewardship to shared leadership, ensuring decision-making authority and accountability is co-owned with those with lived migration experience.
2. Building Leadership and Research Capacity: Ongoing training, mentorship, and peer-learning opportunities are being developed to equip LEAP and wider network members with skills in research design, grant writing, facilitation, and public engagement. These efforts aim to enable members to lead their own projects, influence research agendas, and hold institutional partners accountable to equitable practice.
3. Securing Long-Term Funding: The MHCRN is building partnerships with NGOs, public health agencies, and academic institutions committed to inclusion health and participatory research. These collaborations aim to diversify funding streams and secure multi-year grant income that recognise the value of community-led engagement beyond short-term project cycles. We have been successful in securing funding for the next three-five years.
4. Institutional Integration and Administrative Reform: The network is working with City St George’s to support the integration of Patient and Public Involvement and Engagement (PPIE), participatory research approaches and peer research roles as routine elements of all research grants through the potential creation of a co-production centre and contributing to the Participatory Research Hub (https://www.staff.sgul.ac.uk/research-support/participatory-research-hub/participatory-research-hub). This includes advocating for administrative reforms such as streamlined systems for paying peer researchers that make participatory approaches practical, equitable, and replicable.
5. Embedding Relationship-Based Practice: Sustaining the MHCRN depends on maintaining authentic relationships built on trust, reciprocity, and respect. Mechanisms such as community check-ins through Zoom potential future use of a community WhatsApp group, shared learning events, and co-authorship processes ensure relationships extend beyond individual projects and funding cycles, reinforcing the long-term social infrastructure of the network.

**Fig. 6:**
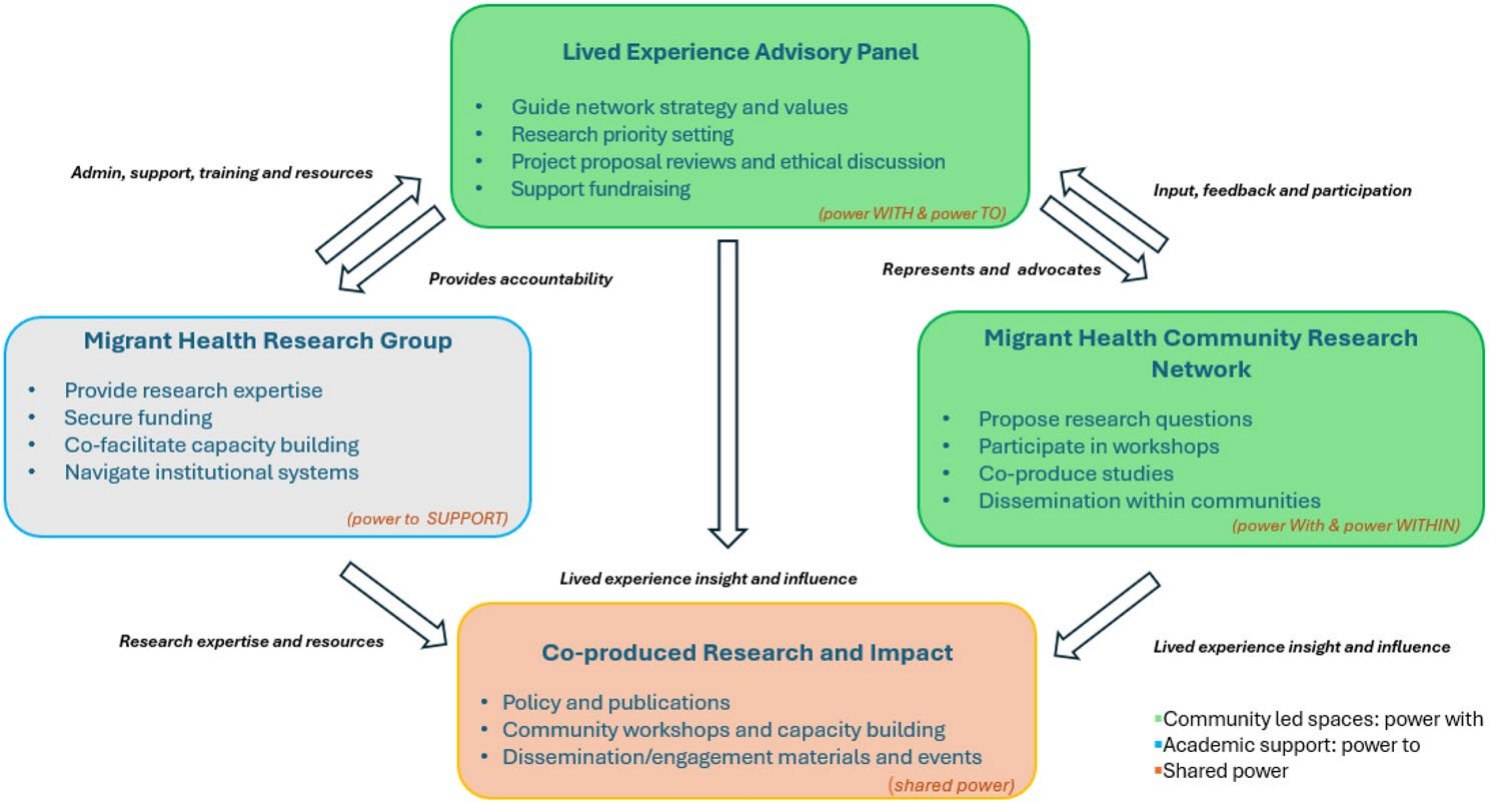
Visual representation of the envisaged interaction between the lived experience advisory panel, migrant health community research network and migrant health research group

As the MHCRN continues to evolve, sustaining equitable partnership requires mechanisms that continually surface and rebalance power dynamics [55]. To support this, we aim to integrate the *Power Wheel* as a reflective and practical tool. The framework distinguishes between *power over* (institutional and academic control), *power to* (the ability to act and influence change), *power with* (collaborative strength built through collective action), and *power within* (confidence and self-belief arising from lived experience) [55]. Below we have created a diagram mapping the interactions, roles and power flow that we are aspiring towards between the MHCRN, LEAP and the MHRG. Applying these concepts will help the network to identify and address both visible and hidden forms of power that shape decision-making, funding, and recognition of expertise. For example, identifying instances of *power over* will enable the group to adjust structures that unintentionally reinforce hierarchy, while building and fostering *power within* will support members to take leadership roles and co-design research agendas [55]. In the future, we hope that using the Power Wheel in workshops and governance meetings will embed critical reflection into our practice, ensuring that decolonisation, equity, and mutual accountability are tangible and sustained commitments rather than aspirational principles Fig. 6.

## Discussion

Our experience developing the MHCRN offers valuable ongoing insights into the challenges and opportunities in creating meaningful academic-community partnerships in migrant health research through a real-world case study with shared learning that can be applicable for other inclusion health groups. These findings and reflections both align with and contribute to the existing literature on community engagement in health research and what “success” in this field could look like [56–59].

The importance of trust-building through acknowledgment of and transparency about past limitations and academic shortcomings mirrors findings from other community-based participatory research initiatives [60]. Other participatory studies have similarly found that transparency and open discussion about current and past research practices helped establish more equitable partnerships with marginalised communities. For example, a study working with survivors of intimate partner violence reflects that the trust-building exercise for their advisory group had been facilitated by transparency and academic partners being honest about their experience of participatory research [61]. Our experience suggests that this approach is particularly crucial for migrant health research, where past experiences of discrimination and exploitation particularly in the health-related research which has been rooted in colonialisation may create additional barriers to trust but discussing these issues openly allows for shared learning and progress.

The structural and sustainability barriers we encountered within academic institutions echo challenges documented across similar initiatives [59]. A recent scoping review of community engagement in areas of poor health and disadvantage found that these structural barriers including short funding cycles, sustained academic staffing to facilitate engagement and the ability for academic institutions to be flexible with regards accessibility seriously hindered participation [59]. However, our experience with the MHCRN suggests these challenges are amplified when working with migrant communities due to additional complexities around immigration status and right to work considerations. The sustainability challenges we encountered reflect broader issues in community-based participatory research. MacFarlane et al. noted similar tensions between project-based funding cycles and the need for sustained community engagement in refugee health research [39]. Our experience suggests that traditional academic funding mechanisms may be particularly ill-suited to supporting the kind of flexible, long-term engagement needed for meaningful collaboration with migrant communities. As such any funding schemes need to consider these power dynamics and to encourage ownership and sense of belonging provide multi-year sustainable funding support to sustain engagement.

Our learning regarding cultural competence and power dynamics expanded our shared understanding of community engagement in migrant health. Many studies have highlighted the importance of cultural sensitivity in research partnerships, our experience with the MHCRN reveals how extreme diversity within migrant communities could disrupt traditional power dynamics by preventing the formation of dominant in-groups with the shared experience being that of lived experience of migration. However, it has also been important to be aware and reflective of different power dynamics to ensure we create a safe space for all. Noting the different lived experiences of network members as well as intersecting identities that may contribute to experiences of power or oppression (e.g. language, ethnicity, country of origin, length of time in the UK, age, gender, sexual orientation), we co-developed norms and expected behaviours of the group (which are referred to in each meeting). We use these norms, facilitators with lived experience of migration and a range of discussion methodologies to support the creation of a safe and supportive space where all feel comfortable sharing.

Through the lens of intersectionality, we also consider migrant groups who are underrepresented in our network (particularly migrants from Eastern Europe, migrants with lower levels of English, migrants with a disability) and we are exploring ways to actively reach out to and facilitate events that are inclusive of these groups. This has included outreach to specific community groups, events which focus on small group engagement with simultaneous translation in multiple languages, a mixture of in-person and online events and ensuring that in-person events are held in an accessible space. While previous research has often treated migrant communities as relatively homogeneous groups, our experience demonstrates the importance of recognising and navigating multiple layers of cultural, social, and power relationships. This complexity requires a more nuanced approach to community engagement than is often acknowledged in the literature.

The solutions to the challenges found developed through the MHCRN, particularly around community meeting locations/informal Zoom catchups and practical support align with best practices identified in other successful community-research partnerships [47, 60, 62, 63]. However, our findings extend this work by demonstrating how these practical considerations take on additional significance when working with migrant communities. The provision of childcare and the opportunity to attend sessions with children, for instance, proved not just helpful but essential for enabling participation from certain community members, particularly women with childcare responsibilities who would otherwise have been unable to attend.

Shared leadership, co-facilitation and having more members of the community than academics present at workshops emerged as a particularly important approach which is reflected in the literature. Our experience suggests that having people with lived experience of migration in leadership roles created safe space that enables more authentic participation [64]. As illustrated by our community partners’ reflection, this shared experience creates immediate credibility and trust that might otherwise take considerable time to establish. The community reflected that migrants would like to go to workshops where they have enough representation and where they feel psychologically safe to contribute.

The development of the MHCRN also highlights the importance of institutional change in enabling meaningful community engagement. While much existing literature focuses on project-level strategies for community engagement, our experience suggests that sustainable change requires fundamental shifts in how academic institutions approach community partnerships. This includes rethinking traditional academic hierarchies, developing new mechanisms for recognising and valuing lived experience, and creating more flexible administrative systems.

Our experience with the MHCRN suggests several key implications for researchers and institutions seeking to develop similar initiatives outlined in box 3 below:

**Table:** 

Box 3 Implications for practice and future research
Practical considerations: Institutions need to develop more flexible administrative systems that can accommodate community partnership needs, including appropriate payment mechanisms and recognition of lived experience expertise. This may require advocacy for policy changes at both institutional and national levels. Researchers should consider investing significant time in relationship-building focusing on genuine and deep understanding of partners’ needs and previous experiences and aiming to develop a shared understanding and approach to working together before pursuing specific research objectives. While this may seem inefficient from a traditional academic perspective, our experience suggests it is essential for developing genuine partnerships. Practical support for participation (such as childcare, accessible venues, travel costs, reimbursement and translation) should be considered core project costs rather than optional extras. These supports are often essential for enabling diverse participation, particularly from more marginalised community members.
Future Directions Exploration of sustainable funding models that can support long-term community engagement beyond traditional project cycles Development of frameworks for evaluating the impact of community-academic partnerships in migrant health research Investigation of how institutional policies and practices can better support community engagement, particularly with migrant communities Understanding how to effectively navigate and leverage the diversity within migrant communities while ensuring inclusive representation

## Conclusions

The development of the MHCRN demonstrates both the possibilities and challenges of creating genuine academic-community partnerships in migrant health research. While significant challenges remain, particularly around institutional structures and sustainable funding, our experience shows that careful attention to process, relationship-building, and power dynamics can help create more equitable and meaningful research partnerships. The network’s evolution offers valuable lessons for others seeking to develop similar initiatives, while also highlighting areas requiring further development and research. Perhaps most importantly, it demonstrates that while creating genuine community-academic partnerships is complex and time-consuming, it is both possible and essential for improving the relevance and impact of migrant health research. The MHCRN continues to evolve, learning from both successes and challenges. Our experience suggests that the future of migrant health research must lie in approaches that genuinely place migrant voices at the centre of research design and implementation, supported by institutional structures that enable rather than hinder such collaboration.

## Data Availability

No datasets were generated or analysed during the current study.
